# Changes in multidisciplinary team decisions in a high volume head and neck oncological center following those made in its preferred partner

**DOI:** 10.3389/fonc.2023.1205224

**Published:** 2023-09-01

**Authors:** Jan-Jaap Hendrickx, Tommy Mennega, Jeroen M. Uppelschoten, C. René Leemans

**Affiliations:** ^1^ Department of Otolaryngology-Head and Neck Surgery, Amsterdam University Medical Center-location VUmc, Amsterdam, Netherlands; ^2^ Department of Radiation Oncology, Noord West Clinic Alkmaar, Alkmaar, Netherlands

**Keywords:** head and neck neoplasms, neoplasm staging, humans surgical oncology, chemoradiotherapy, multidisciplinary team decisions, value based health care, radiotherapy

## Abstract

**Objective:**

Head and neck cancer care is highly complex, and multidisciplinary team meetings (MDTs) are vital for improved outcomes. In the Netherlands, head and neck cancer care is practiced in eight high-volume head and neck oncologic centers (HNOC) and six affiliated hospitals preferred partner (PP) centers. Patients treated in the PP are presented and discussed in the HNOC. To evaluate the importance of these mandatory and decisive steps in decision making, we have assessed the changes in treatment.

**Materials and methods:**

Retrospective evaluation of head and neck cancer patients referred between January 2011 and October 2018 for a MDT evaluation to the HNOC was conducted. The differences in MDT recommendation were classified with regards to major and minor changes.

**Results:**

Management recommendation(MR) changed after 113 of 515 MDT discussions within the PP (487 patients; 22%), of which 86 cases (16%) were major changes. In 67 cases (59.3%), escalation of management was recommended, while in 43 cases (38.1%) de-escalation was recommended.

**Conclusion:**

There was a high rate of change of MRs, when comparing the PP recommendations with the HNOC recommendations. Since patient and tumor characteristics seem unable to predict these changes, we recommend all patients be seen for a clinical presentation, revision of diagnostics, and MDT discussion in a high volume HNOC.

## 1 Introduction

The global burden of cancer worldwide is extensive. The GLOBOCAN (1) estimated an incidence of 18.1 million new cancer cases and 9.6 million cancer deaths. Head and Neck Cancer(HNC) is ranked as fifth among the most common malignancies worldwide, with over 500,000 cases every year (2, 3) HNC arises in the following anatomical sites: oral cavity, oropharynx, hypopharynx and nasopharynx, larynx, nasal cavity and paranasal sinuses, and salivary glands. The most frequent histological type of malignancy in HNC is the squamous cell carcinoma. The risk factors include smoking, excessive alcohol consumption, and infection with high-risk human papillomavirus (HPV). Head and neck tumors and their treatment have a profound impact on the patients’ quality of life, they can affect vital functions such as swallowing, speech, and breathing (4). Workup, diagnosis and treatment decisions are challenging because of the heterogeneity and complexity of HNCs and patients factors (5, 6). The multidisciplinary approach in the management of HNC via multidisciplinary team meetings (MDTs) has proven to be beneficial. MDTs improves survival (7, 8), quality of life (9), preservation of organ-function and change in diagnosis and management (10–12).

In the Netherlands, HNC has been centralized since 1993 in eight Head and Neck Oncologic Centers (HNOC) cooperating within the Dutch Head and Neck Society (DHNS). In close collaboration with the healthcare inspectorate and insurance companies, further guidelines were established to centralize this care in 2011 (13). To increase the quality of care and to distribute the workload, the HNOCs were allowed to work together with a Preferred Partner (PP) center under their supervision. The HNOCs need to treat a minimum of 200 new patients each year PPs need, whereas this number is 80 for PPs. The Amsterdam University Medical Center head and neck oncological center (HNOC) collaborates with Noord West Ziekenhuis Alkmaar as its PP center. In the PP the diagnostic workup is performed for patients presented there, and a weekly MDT is held. All patients diagnosed in the PP center are referred for evaluation to the HNOC. There a thorough history is taken and physical examination is performed. Diagnostic workup and recommended management are subsequently reviewed during a MDT. The majority of the patients is then treated in the PP. Patients with a nasopharyngeal carcinoma, skull base malignancies or those in need of more specialized surgery (sentinel node procedures, transoral robotic surgery and total laryngectomy) are treated in the HHOC.

There is limited scientific evidence addressing the value of evaluation by a higher volume MDT. This retrospective study is a first study that investigate the value of such re-evaluation by comparing changes in Management Recommendation (MR) determined through physical re-evaluation, revision of diagnostic workup and re-classification compared to the original findings.

## 2 Materials and methods

We performed a retrospective analysis of HNC patients referred by the PP to the HNOC in our part of the country, between January 2011 and October 2018. Inclusion criteria were that the patients were discussed in both the MDT of the PP, and in the HNOC-MDT. Patients with an confirm distant metastases were not routinely referred to the HNC. Furthermore, patients had to have a malignant head and neck tumor. The exclusion criteria were incomplete data (n = 17), unspecific MR (n = 10), referral for definitive treatment in the HNOC (n = 7), or patients suffering from a benign head and neck tumor (N-= 6). Treatment takeover was requested, for example, for patients with nasopharyngeal carcinomas or those requiring partial- or total-laryngectomy.

Data collected included patient and disease characteristics, including gender, age, tumor site, type of disease (i.e. primary and recurrent), TNM-classification (according to the UICC TNM 7 and 8 editions (14, 15), comorbidities using the Adult Comorbidity Evaluation 27 (ACE-27) (16) and tumor incidence date. We collected outcome data, including diagnostic workup (i.e. radiology, pathology and physical examination reports) and MRs. Patients with recurrent or second primary cancers were included after repeated presentation to both MDTs.

The main outcome was a change in MRs resulting from the HNOC MDT re-evaluation, classified as major or minor. The major and minor criteria in our study resulted from an adaptation of the criteria described by Brunner et al. (17) (see **Table 1**). Major changes were defined as switching to a different management modality, additions/omissions with great impact on morbidity or within management modality with great impact on morbidity. Minor changes were defined as changes within management modality with small impact on morbidity.

**Table 1:** Classification of changes in treatment modality in major and minor, and absolute number of MDT decisions.

Treatment modality	Major change		n	Minor change		n
Surgery	Neck dissection	Unilateral – Bilateral	3	Neck dissection	add – subtract level	7
	Reconstruction type	Local – Free	10	Reconstruction type	Fibula – Scapula	11
	Resection type	Marginal – Segmental	9			
		TOE – Composite resection	6			
	Change of a primary modality		24			
Radiation therapy	Radiation field	Local – Locoregional	6	Radiation Dosage		5
		Unilateral – Bilateral	1			
		Fraction schedule	1			
	Change of primary modality or addition of a modality	Surgery - Radiotherapy	16			
Chemotherapy	Add – subtract systemic therapy		15	Dosage		4
				Cisplatinum – Cetuximab		

N.A, Not available; TOE, Trans Oral Excision.

In the case of multiple MRs by the PP, a major change was a switch to a recommendation that was not proposed. A minor change was choosing the alternative recommendation, only if an evident primary and alternative recommendations were stated. No change was registered if two comparable recommendations were stated or when in doubt. Change in TNM-classification or additional diagnostic workup without change of treatment strategy was registered as a separate minor change.

Final management (de-)intensification compared to the one by the PP was recorded based on an adaptation of criteria previously described (12) (see **Table 2**). De-intensification was defined as the omission of a treatment modality or a change to a different treatment modality or within a modality with decreased morbidity. Intensification was defined as the addition of a treatment modality or a change to a different treatment modality or within a modality with increased morbidity.

**Table 2:** Classification of (de-) Intensification for the different treatment modalities.

Treatment modality	Intensification	De-intensification
Surgery	Unilateral → bilateral neck dissection	Reconstruction free flap → local
Radiation therapy	Local → loco regional RT	Omission of postoperative radiation
Chemotherapy	Addition of TPF induction	Omission of chemo in concomitant therapy

RT, Radiotherapy; TPF, Docetaxel, cisplatin and Fluorouracil.

To assess the origin of the MR, and to identify specific patient or tumor characteristics changes in in TNM-classification (adjusted classification modality, up- versus down-classification), diagnostic physical examination or diagnostic workup (histopathology, diagnostic imaging) with clinical impact on the MR were also scored.

### 2.1 MDTs setup

The PP NWZ Alkmaar, has a weekly clinic and MDT for HNC patients. The new patients are examined by a head-neck surgeon (otolaryngologist and/or maxillofacial surgeon) and a radiation oncologist. A dermatologist is consulted when necessary. The diagnostic workup was usually performed within two weeks and the patients returned to the clinic for results, after discussion with their MDT. The PP has the same equipment as the HNOC and used the HNOC imaging protocols throughout the study. The patients were referred to the HNOC, during the following week.

The Head and Neck Oncological Center, Amsterdam UMC, location VUMC, has a weekly clinic and MDT for HNC patients. All available information was reviewed, and additional information was gained. All radiologic studies are send to the HNOC, for formal re-evaluation by the head and neck radiologists of the HNOC. The patients are seen in the outpatient clinic, where a complete history is done. Standard head and neck examination includes: inspection of the oral cavity and pharynx bimanual palpation of the mouth, palpation of the neck, (in)direct laryngoscopy and stroboscopy (in patients with a small laryngeal carcinoma).

A management proposal was formulated prior to this MDT by the PP MDT. During the MDT, head and neck surgeons, radiation and medical oncologists were present, besides the radiologists and pathologists, other physicians on indication and supporting staff. The PPs head and neck surgeons and radiation oncologist are attending the HNOC MDT via teleconference. During the MDT discussions MR were based on the current NCCN and national guidelines. Management recommendations are approved or changed and reported back to the PPs physicians. The final MR of the HNOC MDT was usually carried out in the PP, Patients with a nasopharyngeal carcinoma or a skull base malignancy were treated in the HNOC, due the relative rarity in our population. The PP has the possibility to perform many head and neck surgical procedures including microlaryngeal laser surgery, microvascular reconstructions, and to treat patients with (chemo)radiotherapy. In the time frame of the present study the surgeons at the PP, where trained to perform sentinel node procedures. Therefore in the first 6 years of the study patients were referred to the HNOC for treatment, and in the latter 3 years patients were treated in the PP. Total laryngectomies are only performed at the HNOC.

### 2.2 Data collection and statistical analysis

After gaining approval of the institutional review boards in the HNOC, Amsterdam UMC, location VUMC, data was collected and scored using Microsoft Excel (Microsoft, Redmond, Washington). Scoring was done by three independent individuals (JJHT, TM and JMU), of which two are senior attendees of the HNOC MDT (JJH and JMU) to reduce bias. Statistical analysis was performed using SPSS version 22.0 (IBM Corporation, Armonk, New York). Descriptive statistics were used for all data. To compare categorical data, the Chi-square or Fischer’s exact test were used, where a value of p < 0.05 was considered statistically significant.

## 3 Results

### 3.1 Number of patients discussed in both the PP and the HNOC-MDT

**Figure 1:**
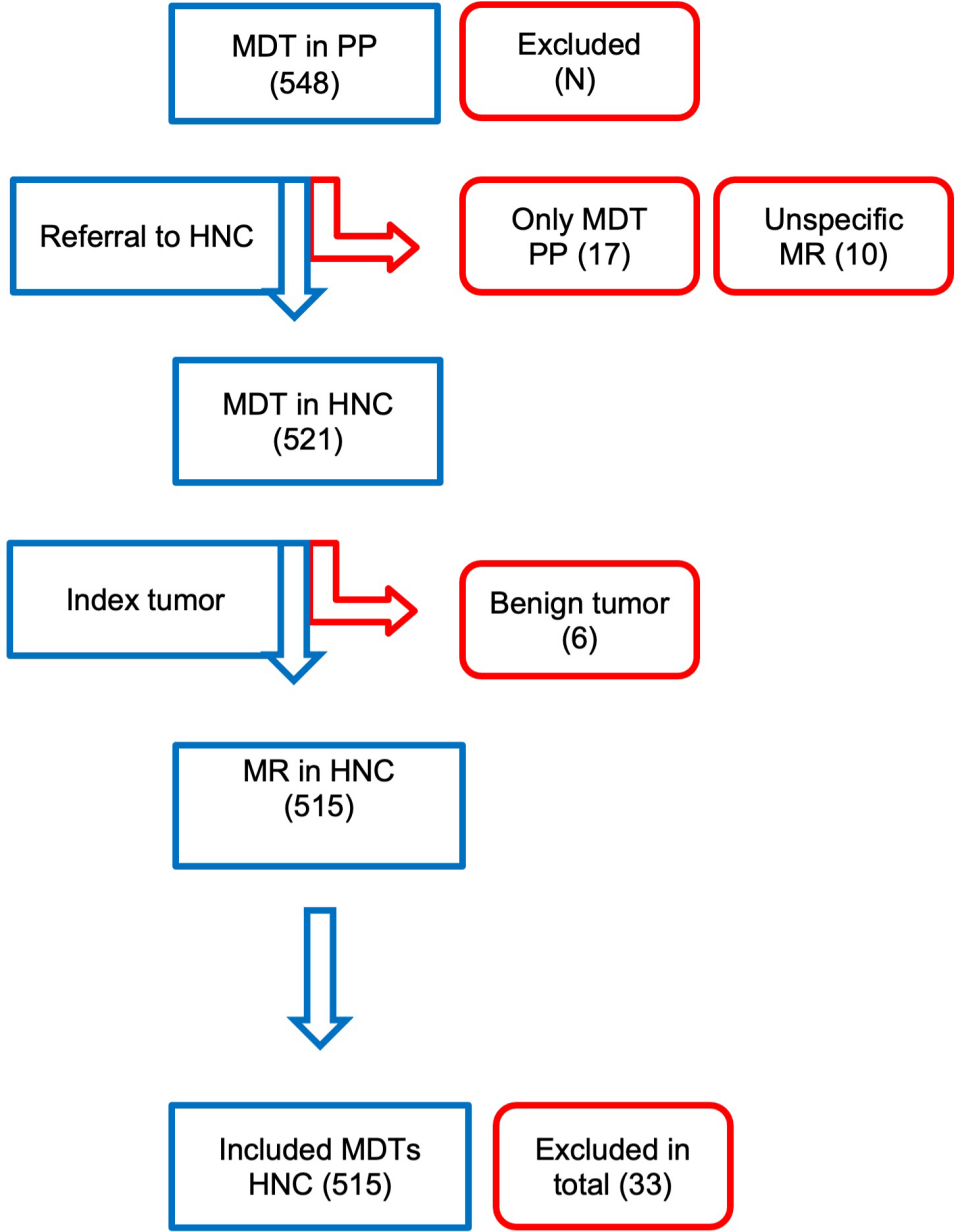
Visual representation on the number of patients referred by the PP, and reasons for exclusion eg. only discussion in PP-MDT, unspecific MR by the PP, patients with benign conditions.

Between January 2011 and October 2018, 548 patients were referred by the PP to the HNOC. In the weekly HNOC-MDT, 10-15 new patients were discussed each week; therefore, during this period, the HNOC-MDT discussed approximately 4500 patients. Thirty-three patients were excluded due to the following reasons: only discussion in the PP-MDT (n = 17), unspecific MR by the PP (n = 10), patients referred with benign head and neck tumors (n = 6) (see **Figure 1**). Twenty-seven patients were referred more than once; 25 patients among them were referred twice, and one patient was referred three times. Resulting in 515 evaluable MDT recommendations.

### 3.2 Descriptive data

The clinical data is summarized in **Table 3**. The mean age of the patients at the time of the MDT was 65 years, and 65% of the patients were male. The most common tumor sites were the oral cavity (n = 185), larynx (n = 129), oropharynx (n = 89), hypopharynx (n = 29), nasal cavity (n = 27), salivary glands (n = 23), unknown primary tumor (n = 19), nasopharynx (n = 3), and others (n = 11). The tumor sites defined as ‘other’, included five cases of cutaneous squamous cell carcinomas and a cutaneous melanoma, squamous cell carcinoma of the external auditory canal, cutaneous sarcoma, Merkel cell carcinoma, recurrent papillary thyroid cancer with tracheal extension and a neck metastasis of a cutaneous squamous cell carcinoma. Over 85% of the cases were primary tumors. Recurrent or residual tumors were seen in 7% of cases and second primary tumors were discussed in 5.6% of the cases. There were no cases with a distant metastatic tumor.

**Table 3:** Patients and tumor characteristics referred by PP, for evaluation in the HNOC-MDT represented in absolute number and percentage of the total.

Variable		Mean in y (min max)
Age		65 (15-92)
Gender		n (%)
	Male	335 (65%)
	Female	180 (35%)
Tumor site		n (%)
	Oral cavity	185 (35.9%)
	Larynx	129 (25%)
	Oropharynx	89 (17.3%)
	Hypopharynx	29 (5.6%)
	Nasal cavity	27 (5.2%)
	Salivary glands	23 (4.5%)
	Unknown primary	19 (3.7%)
	Nasopharynx	3 (0.6%)
	Other	11 (2.1%)
Disease		n (%)
	Primary	450 (87.4%)
	Second primary	29 (5.6%)
	Recurrent/residual	36 (7%)
Overall comorbidity score (ACE-27)		n (%)
	0 (None)	154 (29.9%)
	1 (Mild)	197 (38.3%)
	2 (Moderate)	97 (18.8%)
	3 (Severe)	67 (13%)
T-classification		n (%)
	Tx	0 (0%)
	T0	18 (3.5%)
	Tis	7 (1.4%)
	T1	137 (26.6%)
	T2	116 (22.5%)
	T3	76 (14.8%)
	T4	115 (22.3%)
N-classification		n (%)
	Nx	8 (1.6%)
	N0	312 (60.6%)
	N1	48 (9.3%)
	N2	90 (17.5%)
	N3	11 (2.1%)
M-classification		
	Mx	9 (1.7%)
	M0	460 (89.3%)
	M1	0 (0%)
	Missing	46 (8,9%)

TNM-classification was missing in 46 cases (14 primary, 3 second primary, 25 recurrent and 4 residual tumors).

The most commonly recommended treatment by the PP, was surgery with or without adjuvant treatment based on subsequent histopathological findings (n = 172), followed by primary radiotherapy (n = 114), surgery with planned adjuvant radiotherapy (n = 83), chemoradiotherapy (n = 95) and chemotherapy (n = 1). Some patients of the PP were referred with two or more MRs, eg. Primary chemo-radiotherapy or upfront neck-dissection with adjuvant (chemo-)radiotherapy (n = 50). During the study population several studies were conducted in both the PP and in the HNOC. Inclusion in studies, only ongoing in the HNOC, were not scored as a MR-change.

### 3.3 Changes in management recommendations

Change in MR occurred in 113 cases (22%) and major changes occurred in 86 cases (16.7%). In 67 (59%) of these cases, more intense treatment was recommended (e.g. performing a bilateral neck dissection, where a unilateral neck dissection was proposed). In 43 cases (38%), de-intensification (e.g. only radiotherapy, where concomitant chemotherapy was proposed and in three cases (2%) a different recommendation with the same intensity was proposed. In 63 (12.2%) of the cases the HNOC-MDT advised to perform additional diagnostics e.g. a second ultrasound-guided fine needle aspiration to assess possible regional metastasis. However these recommendations did not result in a change in MR.

Likewise in 63 cases (12.2%), there were minor recommendations that caused changes in tumor re-classification, without an change in MR. E.g. the change from a cT1 oral cavity to an cT2 oral cavity, for which still a transoral excision and sentinel node procedure was recommended.

In 49 cases, imaging review caused minor changes in the recommendation and in the case of 19 patients, this was due to histopathology review. See **Table 4** for details.

**Table 4:** Changes in management recommendations in absolute numbers and percentage of the total.

Change in recommendation		n (%)
Change in recommendation (both major and minor)		113 (22%)
Major change in recommendation		86 (16.7%)
	Surgery	52 (10.1%)
	Radiotherapy	24 (4.7%)
	Chemotherapy	15 (2.9%)
Minor change in recommendation		27 (5.2%)
	Surgery	18 (3.5%)
	Radiotherapy	5 (1.0%)
	Chemotherapy	4 (0.8%)
Minor recommendations (additional diagnostics/no MR change)		63 (12.2%)
Minor recommendations (tumor reclassification/no MR change)		63 (12.2%)
Intensification		67 (59.3%)
De-intensification		43 (38.1%)
Total (major, minor and diagnostics/re-classification)		239 (46.4%)

#### 3.3.1 Subgroup analysis

Subgroup analysis (incidence year, comorbidities, primary versus second primary versus recurrence tumors, tumor site, TNM-classification and proposed management strategy by the PP) revealed no significant differences in prevalence of changes in treatment recommendations by the HNOC-MDT. See the **Supplementary Tables** for further details.

### 3.4 Effect of physical examination or diagnostics workup

Physical reexamination changed MR in 21 cases (4%) (see **Table 5**). Major changes occurred in 18 cases (3.5%) Adjusted classification following physical examination occurred in 22 cases (4%), and histopathology samples were reviewed in 231 cases (45%) by dedicated head and neck pathologists. In 21 cases, the histopathology diagnosis changed after revision (9.1%). This change of diagnosis in 11 cases (52%) had a direct impact on the MR. Additional histopathology was requested in 38 cases by the HNOC. In the HNOC, a dedicated head and neck radiologist revised the diagnostic imaging. Changes in imaging reports were found in 129 cases (25%). These changes in 37 cases (28%) had a direct impact on the recommended management. The TNM-classification was adjusted in 95 cases (21%) after the HNOC MDT, and this was most frequently done because of changes in diagnostic imaging reports (71%) and diagnostic physical evaluations (23%) (see **Table 6**), 63% were down-classified. In 32 cases (15% of total number of cases discussed) both TNM-classification and MR changed. Additional imaging was requested in 93 cases (18%). In 45 cases (8.7%), an additional endoscopy under anesthesia was performed in the HNOC.

**Table 5:** Changes related to diagnostic workup.

Diagnostic physical re-examination	Change MR	n (%)
		21 (4.1%)
Histopathology		
	Revised	231 (45%)
	Change	21 (4.1%)
	Additional	38 (7.4%)
	Change MR	11 (2.1%)
Diagnostic imaging		
	Change	129 (25%)
	Additional	93 (18.1%)
	Change MR	37 (7.2%)
Additional examination under general anesthesia		45 (8.7%)

Changes due to physical reevaluation, histopathological revision, diagnostic imaging reviewing, and additional endoscopy under general anaesthesia.

MR, Management Recommendation.

Changes due to physical reevaluation, histopathological revision, diagnostic imaging reviewing, and additional endoscopy under general anaesthesia.

**Table 6:** Modality responsible for changes.

		N (%)
Change in classification		95 (21.2%)
Up-classification		35 (36.8%)
Modality responsible for change		
	Clinical	22 (23.1%)
	Radiological	68 (71.6%)
	Pathological	5 (5.3%)

Modality, clinical re-examination, radiology revision and pathology revision.

## 4 Discussion

As the literature pointed out, the MDT plays an essential role in the complex management of HNC. Several studies have demonstrated the beneficial aspects of MDTs in terms of treatment and outcome improvement (7–9, 12, 18). National regulations dictates that all HNC patients of the PP have to be evaluated by the HNOC (19, 20). However, to date, no study has shown the effect of this collaboration on the improvement of patient care. Our study showed the significant role of the MDT in this collaboration between the HNOC and PP for the management of HNC patients.

### 4.1 Changes in management recommendation in general

Almost half (46%) of the cases referred to the HNOC had a change in either diagnostics and/or MR. Changes in MR after the HNOC-MDT were found in 22% of the cases, with major changes in 16% of the cases, such as bilateral instead of one-sided neck dissections or the addition of a treatment modality such as (chemo)radiotherapy. The rate of changes in MR during MDT’s in other oncologic fields show a wide range in the literature, ranging from 2% up to 27% (19, 21–23). These differences could be attributed to differences in the validity of pre-MDT recommendation as well as the differences in the details of the recommendation studied (e.g. modality only, drug type, dosage, etc).

A similar collaboration between the University Medical Center Groningen (HNOC) and the Medial Center Leeuwarden (PP) was investigated (24). In this prospective study, eight cases out of the 336 cases (2%) involved a change of recommendation. It should, however, be noted that the patients were presented via video-conferencing. Whereas patients were physically examined in the HNOC in the present study. Furthermore, the radiologic investigations were presented via video-conferencing, whereas the radiologic investigations were completely transferred and formally re-evaluated in our collaboration. Additionally, the pathology slides were revised on indication only. In our series there was no selection bias since all patients were re-evaluated.

The investigated outcomes were changes in MR. The present study does not allow for oncological outcome analysis since all patients were treated according to the recommendations of the HNOC-MDT. Making comparison of patient with and without changes in recommendation difficult to interpret. Comparing oncological results of the current population with those from the literature or national databases was thought to be problematic because of different patient populations and given the fact that the literature usually reports a variability and range in survival.

Treatment intensification is usually instituted with the aim to improve survival, e.g. concomitant chemoradiotherapy vs. Radiotherapy improves survival by 6.5% (25). Moreover treatment deintensification, when appropriate, reduces complications and side-effects, without jeopardizing survival (26). There is considerable evidence that patients treated in centers with a high volume have a better survival (27). These aspects combined suggest that the oncological outcome likely will have been improved by the recommended changes.

There was no association between changes in recommendations and certain subgroups. The implication of this is that all patients may benefit from referral to a high volume MDT.

### 4.2 Effect of pathology revision

Several studies have found that the revising of histopathological slides affects MR. Kronz et al. (28) analysed the literature and found that management changed in 5-7% of the cases because of a second opinion and evaluation of pathology. Westra et al. (29) found that in 7% of the patients, major discrepancies were identified after the second opinion pathology evaluation for HNC. After examining multiple organ systems, a prospective review found that 1.4% of the 6000 cases witnessed a change of diagnosis due to second opinion pathologies (30). Tung et al. (31) found that in 6% of the 715 cases a major change in pathology occurred and in 2% of the total cases, the management was changed after the second opinion pathology. Garcia et al. (32) found that in 20% of cases the revised pathology led to a change in pathology diagnosis. Comparing the aforementioned literature to our study, we found management changes on the basis of revision histopathology in 5% of cases. Since difficult histopathological cases of the PP are sent to the HNOC pathology department for revision independent of the reevaluation in the MDT, this practice likely lead to an artificially low figure.

### 4.3 Effect of diagnostic imaging revision

For diagnostic imaging in HNC, Lysack et al. (33) investigated the impact of second opinion diagnostic imaging review by a dedicated head and neck radiologist. They found an adjusted TNM-classification in 56% of cases, and MR changed in 38% of these patients. Hatzogou et al. (34) found that second-opinions in neuroimaging, resulted in disagreement in 55 (19%)patients, and the management or disease classification was changed in 39 (15%) patients out of 55. Recently the importance of the presence of an head and neck radiologist was investigated by Alterio et al. (35), showing that in 52% of the cases there was a change to the initial radiologic report. In 27% the imaging was considered in adequate. And in 25% there was a modification in tumor staging or in MR. When comparing this with our population, adjustments in classification could be attributed to radiology solely in 25% of the cases. However, this resulted in a change of MR in only 7% of the cases. Therefore, the absolute percentage of radiology adjustments aligned with the literature.

### 4.4 Effect on TNM-classification

While examining the effect of the second evaluations on classification, we found changes in 21% of the cases. The study of Wheless et al. (12) found in changes in classification in 12% of cases. Bergamini et al. (18) found reclassification necessary in 49% of the cases, without stating further details. In the second study, requests for better classification were registered instead of actual changes. Explanations for the change in classification could be the time interval between examinations, combined with the rate of progression (36). Although the time between the two MDT recommendations was not measured in the present study, the interval was likely to a maximum of 3 weeks since national guidelines were strictly adhered to.

### 4.5 Limitations

The current study was retrospective in by nature, which was both its strength as well as its limitation. As such there was no control group without the HNOC-MDT. Therefore a direct comparison fo the HNOC-MDT influence on outcome was not possible.

Furthermore the retrospective nature, caused difficulties to determine the relative effect of physical examination/pathology/radiology on the changes in MR.

In this study, the HNOC-MDT is considered the gold standard. This is based on the higher volume of patients and superior experience of the team members.

## 5 Conclusions

In conclusion, our study explores the impact of the HNOC MDT in managing HNC patients. Changes were found in 22% of the cases, of which 17% were major changes. Changes were due to both physical examination and radiological image review. In this study no information could be obtained on impact of changes on functional outcome and survival. No subgroup of patients was exempt of a changed management. Therefore, we advocate that patients treated in low-volume centers should be presented in an high-volume HNOCs to confirm or adjust the treatment.

## Data availability statement

The raw data supporting the conclusions of this article will be made available by the authors, without undue reservation.

## Ethics statement

Ethical review and approval was not required for the study on human participants in accordance with the local legislation and institutional requirements. Written informed consent for participation was not required for this study in accordance with the national legislation and the institutional requirements.

## Author contributions

JJH, JU and CLE conceived of the presented idea. JJH and TM developed the theory and methodological framework. TM collected the data, JJH and JU supervised the data collection. TM and JJH verified the analytical methods. All authors contributed to the article and approved the submitted version.
