# Supplementary material for: Changes in multidisciplinary team decisions in a high volume head and neck oncological center following those made in its preferred partner
Source: Front Oncol. 2023 Sep 1;13:1205224. doi: 10.3389/fonc.2023.1205224 (PMC10505803; doi:10.3389/fonc.2023.1205224)
Supplement: Supplementary file 1 [file Table_1.docx]

Supplementary Material

Multidisciplinary team decisions in a high volume head and neck oncological center following those made in its preferred partner

**Jan-Jaap Hendrickx ^1^*, Tommy Mennega^1^, Jeroen M. Uppelschoten^2^, C. René Leemans^1^**

*** Correspondence:** Corresponding Author j.hendrickx@amsterdamumc.nl

# Supplementary Data

**Subgroup analyses**

Table 1. Change in Management recommendation by Year of presentation. (*p =* 0.147)

|  | | | Change in management recommendation | | Total | Major |
| --- | --- | --- | --- | --- | --- | --- |
|  |  |  | No | Yes |  |  |
| Years | 2011 | Count | 39 | 12 | 51 | 9 |
|  |  | % | 76.5% | 23.5% | 100.0% |  |
|  | 2012 | Count | 45 | 17 | 62 | 15 |
|  |  | % | 72.6% | 27.4% | 100.0% |  |
|  | 2013 | Count | 50 | 18 | 68 | 13 |
|  |  | % | 73.5% | 26.5% | 100.0% |  |
|  | 2014 | Count | 52 | 23 | 75 | 17 |
|  |  | % | 69.3% | 30.7% | 100.0% |  |
|  | 2015 | Count | 58 | 9 | 67 | 6 |
|  |  | % | 86.6% | 13.4% | 100.0% |  |
|  | 2016 | Count | 48 | 11 | 59 | 7 |
|  |  | % | 81.4% | 18.6% | 100.0% |  |
|  | 2017 | Count | 66 | 16 | 82 | 14 |
|  |  | % | 80.5% | 19.5% | 100.0% |  |
|  | 2018 | Count | 44 | 7 | 51 | 5 |
|  |  | % | 86.3% | 13.7% | 100.0% |  |
| Total | | Count | 402 | 113 | 515 | 86 |
|  |  | % | 78.1% | 21.9% | 100.0% |  |

Table 2. Change in Management recommendation by Classifications of Comorbidity (*p =* 0.340)

|  | | | Change in management recommendation | | Total | Major |
| --- | --- | --- | --- | --- | --- | --- |
|  |  |  | No | Yes |  |  |
| Classification of Comorbidity (ACE-27) | 0 | Count | 126 | 28 | 154 | 23 |
|  |  | % | 81.8% | 18.2% | 100.0% |  |
|  | 1 | Count | 146 | 51 | 197 | 37 |
|  |  | % | 74.1% | 25.9% | 100.0% |  |
|  | 2 | Count | 78 | 19 | 97 | 14 |
|  |  | % | 80.4% | 19.6% | 100.0% |  |
|  | 3 | Count | 52 | 15 | 67 | 12 |
|  |  | % | 77.6% | 22.4% | 100.0% |  |
| Total | | Count | 402 | 113 | 515 | 86 |
|  |  | % | 78.1% | 21.9% | 100.0% |  |

Table 3. Change in Management recommendation by Tumor types (*p =* 0.415)

|  | | | Change in management recommendation | | Total | Major |
| --- | --- | --- | --- | --- | --- | --- |
|  |  |  | No | Yes |  |  |
| Tumor type | Primary | Count | 350 | 100 | 450 | 78 |
|  |  | % | 77.8% | 22.2% | 100.0% |  |
|  | Second primary | Count | 23 | 6 | 29 | 3 |
|  |  | % | 79.3% | 20.7% | 100.0% |  |
|  | Residu | Count | 2 | 2 | 4 | 2 |
|  |  | % | 50.0% | 50.0% | 100.0% |  |
|  | Recurrence | Count | 27 | 5 | 32 | 3 |
|  |  | % | 84.4% | 15.6% | 100.0% |  |
| Total | | Count | 402 | 113 | 515 | 86 |
|  |  | % | 78.1% | 21.9% | 100.0% |  |

Table 4. Change in Management recommendation by Tumor sites (*p =* 0.295)

|  | | | Change in management recommendation | | Total | Major |
| --- | --- | --- | --- | --- | --- | --- |
|  |  |  | No | Yes |  |  |
| Tumor site | Oral cavity | Count | 141 | 44 | 185 | 33 |
|  |  | % | 76.2% | 23.8% | 100.0% |  |
|  | Other | Count | 8 | 3 | 11 | 3 |
|  |  | % | 72.7% | 27.3% | 100.0% |  |
|  | Oropharynx | Count | 69 | 20 | 89 | 13 |
|  |  | % | 77.5% | 22.5% | 100.0% |  |
|  | Nasopharynx | Count | 3 | 0 | 3 | 0 |
|  |  | % | 100.0% | 0.0% | 100.0% |  |
|  | Hypopharynx | Count | 20 | 9 | 29 | 8 |
|  |  | % | 69.0% | 31.0% | 100.0% |  |
|  | Larynx | Count | 110 | 19 | 129 | 17 |
|  |  | % | 85.3% | 14.7% | 100.0% |  |
|  | Nasal cavity | Count | 21 | 6 | 27 | 4 |
|  |  | % | 77.8% | 22.2% | 100.0% |  |
|  | Salivary glands | Count | 18 | 5 | 23 | 2 |
|  |  | % | 78.3% | 21.7% | 100.0% |  |
|  | Unknown primary | Count | 12 | 7 | 19 | 6 |
|  |  | % | 63.2% | 36.8% | 100.0% |  |
| Total | | Count | 402 | 113 | 515 | 86 |
|  |  | % | 78.1% | 21.9% | 100.0% |  |

Table 5. Change in Management recommendation by TNM-classification. T: (*p =* 0.115); N: (*p =* 0.706); M: (*p =* 0.668) Missing cases n=46.

|  | | | Change in management recommendation | | Total | Major |
| --- | --- | --- | --- | --- | --- | --- |
|  |  |  | No | Yes |  |  |
| T-Classification | Tis | Count | 4 | 3 | 7 | 2 |
|  |  | % | 57.1% | 42.9% | 100.0% |  |
|  | T0 | Count | 12 | 6 | 18 | 5 |
|  |  | % | 66.7% | 33.3% | 100.0% |  |
|  | T1 | Count | 108 | 29 | 137 | 23 |
|  |  | % | 78.8% | 21.2% | 100.0% |  |
|  | T2 | Count | 82 | 34 | 116 | 25 |
|  |  | % | 70.7% | 29.3% | 100.0% |  |
|  | T3 | Count | 64 | 12 | 76 | 9 |
|  |  | % | 84.2% | 15.8% | 100.0% |  |
|  | T4 | Count | 93 | 22 | 115 | 15 |
|  |  | % | 80.9% | 19.1% | 100.0% |  |
| Total (T) | | Count | 363 | 106 | 469 | 79 |
|  |  | % | 77.4% | 22.6% | 100.0% |  |
| N-classification | Nx | Count | 7 | 1 | 8 | 1 |
|  |  | % | 87.5% | 12.5% | 100.0% |  |
|  | N0 | Count | 238 | 74 | 312 | 57 |
|  |  | % | 76.3% | 23.7% | 100.0% |  |
|  | N1 | Count | 36 | 12 | 48 | 7 |
|  |  | % | 75.0% | 25.0% | 100.0% |  |
|  | N2 | Count | 74 | 16 | 90 | 11 |
|  |  | % | 82.2% | 17.8% | 100.0% |  |
|  | N3 | Count | 8 | 3 | 11 | 3 |
|  |  | % | 72.7% | 27.3% | 100.0% |  |
| Total (N) | | Count | 363 | 106 | 469 | 79 |
|  |  | % | 77.4% | 22.6% | 100.0% |  |
|  | |  |  |  |  |  |
| M-Classification | Mx | Count | 7 | 2 | 9 | 77 |
|  |  | % | 77.8% | 22.2% | 100.0% |  |
|  | M0 | Count | 356 | 104 | 460 | 2 |
|  |  | % | 77.4% | 22.6% | 100.0% |  |
| Total (M) | | Count | 363 | 106 | 469 | 79 |
|  |  | % | 77.4% | 22.6% | 100.0% |  |

Table 6. Change in Management recommendation by Management type (*p =* 0.223)

|  | | | Change in management recommendation | | Total | Major |
| --- | --- | --- | --- | --- | --- | --- |
|  |  |  | No | Yes |  |  |
| Management type | Surgery | Count | 129 | 43 | 172 | 34 |
|  |  | % | 75.0% | 25.0% | 100.0% |  |
|  | Radiotherapy | Count | 93 | 21 | 114 | 18 |
|  |  | % | 81.6% | 18.4% | 100.0% |  |
|  | Chemotherapy | Count | 1 | 0 | 1 | 0 |
|  |  | % | 100.0% | 0.0% | 100.0% |  |
|  | CRT | Count | 81 | 14 | 95 | 11 |
|  |  | % | 85.3% | 14.7% | 100.0% |  |
|  | Surgery + (C)RT | Count | 63 | 20 | 83 | 15 |
|  |  | % | 75.9% | 24.1% | 100.0% |  |
|  | Other | Count | 35 | 15 | 50 | 8 |
|  |  | % | 70.0% | 30.0% | 100.0% |  |
| Total | | Count | 402 | 113 | 515 | 86 |
|  |  | % | 78.1% | 21.9% | 100.0% |  |
